# Resolving elliptic boundary value problem and integral equation in relational partial metric space

**DOI:** 10.1016/j.heliyon.2024.e40766

**Published:** 2024-11-29

**Authors:** Meena Joshi, Anita Tomar, Mohammad Sajid, S.K. Padaliya

**Affiliations:** a L. S. M. Campus, Pithoragarh-262501, Soban Singh Jeena Uttarakhand University, India; b Pt. L. M. S. Campus, Sridev Suman Uttarakhand University, Rishikesh-249201, India; c Department of Mechanical Engineering, College of Engineering, Qassim University, Saudi Arabia; d S.G.R.R. (P.G.) College Dehradun-248001, India

**Keywords:** 47H09, 47H10, 54H25, Fixed point theorem, Elliptic boundary value problem, Partial metric space, Nonlinear equations, Nonlinear system

## Abstract

In the present study, a novel version of the contraction theory on a relational partial metric space associated with a binary relation is exhibited. In this process, we observe that numerous relations can be used in many well-known fixed point theorems earlier introduced by multiple authors. We solve an elliptic boundary value problem and an integral equation to make the significance of the main conclusion evident. Exploring a nonlinear system, governed by nonlinear equations, underscores the importance of comprehending fixed points - pivotal states where the dynamics of a system remain unaltered.

## 1 Introduction

Metric fixed point theory evolved with the perception of successive approximations in the contraction principle proposed by Banach [8] and is one of the fascinating topics of research. The reason behind this is the applications of the obtained conclusions in almost all branches of science. In the analysis of a nonlinear system, which is characterized by nonlinear equations, understanding the fixed points becomes essential as they represent stable states where the dynamics of a system remain unchanged. Researchers and mathematicians engaged in this endeavor navigate the mathematical terrain with precision, striving to unlock the secrets embedded in the relationships between points in these specialized metric spaces (see, [1], [6], [7], [14], [15], [16], [18], [23] and so on). Ćirić [11] utilized the following contraction to determine unique fixed, $d(Tx,Ty)\leq \mu \max\{d(x,y),d(x,Tx),d(y,Ty),\frac{1}{2}[d(x,Ty)+d(y,Tx)]\}$, x,y∈X,μ∈(0,1), while Hardy-Rogers [12] utilized d(Tx,Ty)≤αd(x,y)+βd(x,Tx)+γd(y,Ty)+δd(x,Ty)+ηd(y,Tx), where, α+β+γ+δ+η<1,α,β,γ,δ,η≥0. In fact, both Ćirić [10] and Hardy-Rogers [12] involved all the possible combinations of distances d(Tx,Ty),d(x,y),d(x,Tx),d(y,Ty), d(x,Ty),d(y,Tx) in a linear way. On the other hand, Banach [8] utilized only the first two distances and is a corollary of Ćirić [10] and Hardy-Rogers [12]. Also, conclusions for fixed point theorems of Kannan [13], Chatterjea [9], Reich [24] are corollaries of Hardy-Rogers [12], and all these theorems are corollaries of Ćirić [10].

This current work extends two fascinating fixed-point conclusions Ćirić [11] and Hardy-Rogers [12] to a relational partial metric space without levying a continuity condition on the involved map and completeness of the underlying space. For this, we initiate the use of R-continuity, R-completeness, R-self-closedness, and regularity in a relational partial metric space. Examples and techniques to solve an elliptic boundary value problem and an integral equation are furnished to indicate the genuineness and effectiveness of the established conclusions, which enhance, broaden, and refine some conclusions existing in the literature. In this reference, these contractions are comparatively weak compared to celebrated contractions because these hold only on the points in the involved relation instead of the whole space.

## 2 Preliminaries

First, we discuss the elementary notions and essential terminology needed to comprehend the subsequent discussions. Now, $N_{0}$, N, and R symbolize sets of whole numbers, natural numbers, and real numbers, respectively, and X is a non-empty set. A binary relation R can be characterized as a subset of the cartesian product X×X. If *x* and *y* are both elements of the set X and belong to the relation R, it implies that *x* is in a relationship with *y*.

Definition 2.1 [21] For x,y∈X, if either (x,y)∈R or (y,x)∈R, that is, [x,y]∈R, then R is called complete.

Definition 2.2 [19] The symmetric closure of a relation R is derived as $R^{s}=R⋃R^{-1}$, where, $R^{-1}=\{(x,y):(y,x)\in R\}$ represents the smallest symmetric relation containing R.

Definition 2.3 [2] Define a self-map T on the set X. The binary relation R is T-closed if for any pair of elements

(x,y)∈R⟹(Tx,Ty)∈R,x,y∈X.

Definition 2.4 [2] A sequence $\left\{x_{n}\right\}$ in the set X is considered R-preserving if for every natural number *n*, the pair $(x_{n},x_{n+1})\in R$.

Definition 2.5 [25] Let Y⊆X and for x,y∈Y, we have z∈X and $(x,z),(y,z)\in R^{s}$, then Y is $R^{s}$-directed.

Definition 2.6 [17] If $z_{0}=x,\;z_{k}=y$, then a sequence ${\left\{z_{j}\right\}}_{j=0}^{k}$, where all $z_{j}$ are not necessarily distinct, defined in X is known as a path of length k∈N connecting *x* to *y* in R if $(z_{i},z_{i+1})\in R$, 0≤i≤k−1, that is, a path of length *k* requires the presence of k+1 elements from the set X. A family of paths in R is symbolized by Γ(x,y,R).

Definition 2.7 [3] Let Y⊆X. If a path exists connecting *x* to *y* in R for x,y∈Y, then Y is R-connected.

Definition 2.8 [20] The function $\rho :X\times X\to R^{+}$ qualifies as a partial metric under the following subsequent hypotheses

$\left(\rho_{1}\right)$ ρ(x,x)=ρ(y,y)=ρ(x,y) iff x=y;
$\left(\rho_{2}\right)$ ρ(x,x)≤ρ(x,y);
$\left(\rho_{3}\right)$ ρ(x,y)=ρ(y,x);
$\left(\rho_{4}\right)$ ρ(x,y)≤ρ(x,z)+ρ(z,y)−ρ(z,z),x,y,z∈X.

Definition 2.9 [20] The sequence $\left\{x_{n}\right\}$ in a partial metric space (X,ρ) is

(i) considered Cauchy if $\lim_{m,n\to \infty }\rho (x_{m},x_{n})$ exists finitely;
(ii) considered convergent to x∈X, if $\lim_{n\to \infty }\rho (x_{n},x)=\lim_{m,n\to \infty }\rho (x_{m},x_{n})=\rho (x,x)$;
(iii) (X,ρ) is deemed complete if each Cauchy sequence within the set X converges to a point in X.

## 3 Main results

This section presents our primary findings and key outcomes. Suppose X[T,R] represents the set of points x∈X satisfying (x,Tx)∈R and F(T) symbolizes the collection of fixed points of a self-map T.

We familiarize R-continuity, R-completeness, and R-self-closedness in a partial metric space (X,ρ,R) associated with a binary relation.

Definition 3.1 (see, [3] for the metric version) In the context of (X,ρ,R), a self-map T:X→X is considered to be R-continuous at the point *x* if, when considering an R-preserving sequence $\{x_{n}\}\subseteq X$ such that the limit of $Tx_{n}$ approaches Tx as $x_{n}$ approaches *x*. Moreover, T is deemed R-continuous if it exhibits R-continuity at every point in the set X.

Example 3.1 Let X=[0,10]. Describe a partial metric ρ:X×X→[0,∞) on U as ρ(x,y)=max⁡{x,y},x,y∈X and a binary relation on X is represented as R={(0,0),(0,1),(1,0),(1,1),(1,2),(2,1),(0,2),(2,0),(2,2),(1,3),(3,4),(4,5),(5,6),(7,8),(9,10)}. Take an R-preserving sequence $\left\{x_{n}\right\}$ so that $(x_{n},x_{n+1})\in R$ and $\{x_{n}\}\to x$, $n\in N_{0}$. We observe that $\left(x_{n},x_{n+1})\notin \{(1,3),(3,4),(4,5),(5,6),(7,8),(9,10)\right\}$ and $\left(x_{n},x_{n+1})\in \{(0,0),(0,1),(1,0\right)$, (1,1),(1,2),(2,1),(0,2),(2,0),(2,2)}, $n\in N_{0}$, which means $x_{n}\subseteq \{0,1,2\}$. Now, define map T:X→X as $Tx=\left\{ \begin{array}{cc} 1, & x\in [0,2]\\ 5, & \text{otherwise} \end{array}\right.$. Clearly, T is R-continuous however not continuous.

Definition 3.2 (see, [3] for the metric version) The space (X,ρ,R) is defined as R-complete when every Cauchy sequence that preserves R within the set X converges to a point that belongs to X.

A complete partial metric space is necessarily guaranteed to be R-complete but the reverse implication may not necessarily be true.

Example 3.2 Consider the set X=(0,2]. Let a partial metric on X is represented as ρ(x,y)=max⁡{x,y}, x,y∈X and a binary relation on X is represented as R={(1,1),(1,2),(2,1),(2,2)}. The space (X,ρ,R) is indeed R-complete, however, it is not complete. This is evident because the Cauchy sequence $\left\{\frac{1}{n}\right\}$ within X=(0,2] converges to 0 which is not an element of X. Here, all possible R-preserving Cauchy sequences converge to 1 or 2.

Definition 3.3 (see, [2] for the metric version) If for each R-preserving sequence $\left\{x_{n}\right\}\subseteq X$, where $(x_{n},x_{n+1})\in R$, $n\in N_{0}$ and $x_{n}\to x$, we have a subsequence $\left\{x_{n_{k}}\right\}$ of $\left\{x_{n}\right\}$ satisfying $[x_{n_{k}},x]\in R$ with $n_{k}\in N_{0}$, then a binary relation R is considered to be *ρ*-self-closed.

Example 3.3 Consider the set X=[0,∞). Define a partial metric on X as ρ(x,y)=max⁡{x,y}, x,y∈X and a binary relation on X is represented as $R=\left\{(\frac{1}{n},\frac{1}{n+1}),(0,\frac{1}{2n}),\;n\in N_{0}\right\}$. Here, $\left\{x_{n}\}=\{\frac{1}{n}\right\}$ is an R-preserving sequence such that $\{x_{n}\}\to 0$ and its subsequence $\left\{x_{n_{k}}\}=\{\frac{1}{2n_{k}}:n_{k}\in N\right\}$ satisfying $[x_{n_{k}},0]\in R$. Then, R is *ρ*-self-closed.

Next, we familiarize regularity in (X,ρ,R).

Definition 3.4 (see, [25] for the metric version) If for a sequence $\left\{x_{n}\right\}\subseteq X$, $(x_{n},x_{n+1})\in R^{s}$, n∈N and $x_{n}\to x$, we have a subsequence $\left\{x_{n_{k}}\right\}$ of $\left\{x_{n}\right\}$ so that $(x_{n_{k}},x)\in R^{s}$, $n_{k}\in N_{0}$, then $\left(X,\rho ,R^{s}\right)$ is regular.

With this in hand, we can now demonstrate our primary finding motivated by the understanding that delving into a nonlinear system, characterized by a nonlinear equation highlights the significance of comprehending fixed points - critical states where the dynamics of a system remain unchanged - we establish a unique fixed point in an R-complete partial metric space endowed with a binary relation.

Theorem 3.1 *Consider*(X,ρ,R)*to be an*R*-complete partial metric space associated with a binary relation*R*and*T*to be a self-map on*X*satisfying the subsequent suppositions:*

(*i*) R*is*T*-closed;*
(ii) X[T,R]*is nonempty;*
(iii) *either*R*is ρ-self-closed or*R*-continuous;*
(iv) *there exists*μ∈(0,1)*satisfying*
  (3.1) ρ(Tx,Ty)≤μΔ(x,y),(x,y)∈R,
  *where,*$\Delta (x,y)=\max\{\rho (x,y),\rho (x,Tx),\rho (y,Ty),\frac{1}{2}(\rho (x,Ty)+\rho (yTx))\}$*, then a self-map*T*on*X*possesses a fixed point.*
(*v*) *Also, if*T(X)*is*$R^{s}$*-connected, that is, there exists a nonzero*$\Gamma (x,y,R^{s})\neq 0$*,*

*then*T*possesses a unique fixed point within the set*X*.*

Proof Examine a Picard sequence $\left\{x_{n}\right\}\subseteq X$ so that $x_{n+1}=Tx_{n}$, $n\in N_{0}$, starting from initial point $x_{0}\in X[T,R]$ as X[T,R]≠ϕ. Since $(x_{0},Tx_{0})\in R$ and R is T-closed, $(Tx_{0},T^{2}x_{0}),(T^{2}x_{0},T^{3}x_{0}),\ldots ,(T^{n}x_{0},T^{n+1}x_{0})\in R$. So

$$(x_{n},x_{n+1})\in R,\;n\in N_{0}.$$

That is, $\left\{x_{n}\right\}$ is an R-preserving sequence. Next utilizing inequality (3.1):

$$\rho (x_{n},x_{n+1})=\rho (Tx_{n-1},Tx_{n})\leq \mu \Delta (x_{n-1},x_{n}),\;n\in N_{0},$$

where,

$$\Delta (x_{n-1},x_{n})=\max\{\rho (x_{n-1},x_{n}),\rho (x_{n-1},Tx_{n-1}),\rho (x_{n},Tx_{n}),\frac{1}{2}(\rho (x_{n-1},Tx_{n})+\rho (x_{n},Tx_{n-1}))\}=\max\{\rho (x_{n-1},x_{n}),\rho (x_{n-1},x_{n}),\rho (x_{n},x_{n+1}),\frac{1}{2}(\rho (x_{n-1},x_{n+1})+\rho (x_{n},x_{n}))\}\leq \max\{\rho (x_{n-1},x_{n}),\rho (x_{n},x_{n+1}),\frac{1}{2}(\rho (x_{n-1},x_{n})+\rho (x_{n},x_{n+1}))\}=\max\{\rho (x_{n-1},x_{n}),\rho (x_{n},x_{n+1})\}.$$

Case (i) If $\rho (x_{n-1},x_{n})\leq \rho (x_{n},x_{n+1})$, then $\rho (x_{n},x_{n+1})\leq \mu \rho (x_{n},x_{n+1})$, a contrariety.
Case(ii) If $\rho (x_{n},x_{n+1})\leq \rho (x_{n-1},x_{n})$, then $\rho (x_{n},x_{n+1})\leq \mu \rho (x_{n-1},x_{n})$.

By proceeding

(3.2) $$\rho (x_{n},x_{n+1})\leq \mu^{n}\rho (x_{0},x_{1}).$$

If m≥n, $m,n\in N_{0}$, then

$$\rho (x_{n},x_{m})\leq \rho (x_{n},x_{n+1})+\rho (x_{n+1},x_{n+2})+\ldots +\rho (x_{m-1},x_{m})-(\rho (x_{n+1},x_{n+1})+\rho (x_{n+2},x_{n+2})+\ldots +\rho (x_{m-1},x_{m-1}))\leq \rho (x_{n},x_{n+1})+\rho (x_{n+1},x_{n+2})+\ldots +\rho (x_{m-1},x_{m})=(\mu^{n}+\mu^{n+1}+\ldots +\mu^{m-1})\rho (x_{0},x_{1})=\frac{\mu^{n}(1-\mu^{m-n})}{1-\mu }\rho (x_{0},x_{1})\leq \frac{\mu^{n}}{1-\mu }\rho (x_{0},x_{1})\to 0,\;as\;n\to \infty .$$

Thus, $\left\{x_{n}\right\}$ forms an R-preserving Cauchy sequence in the set X. The R-completeness of the space implies that $x_{n}\to x\in X$.

When T is R-continuous, then $x_{n+1}=Tx_{n}\to Tx$ and Tx=x, hence *x* is a fixed point of T.

Assuming, R to be *ρ*-self-closed. Then for an R-preserving sequence $\left\{x_{n}\right\}$ in X as $x_{n}⟶x$, we have a subsequence $\left\{x_{n_{k}}\right\}$ of $\left\{x_{n}\right\}$ so that $[x_{n_{k}},x]\in R$, $k\in N_{0}$ and $x_{n_{k}}⟶x$. Consider x≠Tx, then

$$\rho (x,Tx)\leq \rho (x,Tx_{n_{k}})+\rho (Tx_{n_{k}},Tx)-\rho (Tx_{n_{k}},Tx_{n_{k}})=\rho (x,x_{n_{k+1}})+\mu \max\{\rho (x_{n_{k}},x),\rho (x_{n_{k}},Tx_{n_{k}}),\rho (x,Tx),\frac{1}{2}(\rho (x_{n_{k}},Tx)+\rho (x,Tx_{n_{k}}))\}-\rho (x_{n_{k+1}},x_{n_{k+1}})\to \rho (x,x)+\mu \max\{\rho (x,x),\rho (x,Tx)\}-\rho (x,x),\;\;\text{as}\;\;n\to \infty ,=\mu \rho (x,Tx),$$

a contradiction. Thus Tx=x. Hence, *x* is a fixed point of a self-map T.

In case F(T) is a singleton, the proof is complete. Suppose, if possible F(T) is not singleton and *x* and $x^{⁎}$ are both fixed points of T, that is, Tx=x and $Tx^{⁎}=x^{⁎}$. So

$$x,\;x^{⁎}\in T(X)\;\;\text{and}\;\;T^{n}x=x,\;\;T^{n}x^{⁎}=x^{⁎},\;\;n\in N_{0}.$$

As T(X) is $R^{s}$-connected, we have a path $\left\{y_{0},\;y_{1},\;y_{2},\;\ldots ,\;y_{k}\right\}$ of length *k* in $R^{s}$ which connect *x* to $x^{⁎}$ (say), therefore $y_{0}=x,\;y_{k}=x^{⁎}$ and $(y_{i},y_{i+1})\in R^{s}$, i=0,1,2,…,k−1. T-closedness of R implies that, $(T^{n}y_{i},T^{n}y_{i+1})\in R^{s}$, $n\in N_{0}$ and 0≤i≤k−1. So

(3.3) $$\rho (x,x^{⁎})=\rho (T^{n}y_{0},T^{n}y_{k})\leq \Sigma_{i=0}^{k}\rho (T^{n}y_{i},T^{n}y_{i+1})-\Sigma_{i=1}^{k-1}\rho (T^{n}y_{i},T^{n}y_{i})\leq \Sigma_{i=0}^{k}\rho (T^{n}y_{i},T^{n}y_{i+1}).$$

(3.4) $$\rho (T^{n}y_{i},T^{n}y_{i+1})\leq \mu \max\{\rho (T^{n-1}y_{i},T^{n-1}y_{i+1}),\rho (T^{n-1}y_{i},T^{n}y_{i+1}),\rho (T^{n}y_{i+1},T^{n}y_{i}),\;\;\;\frac{1}{2}(\rho (T^{n-1}y_{i},T^{n}y_{i})+\rho (T^{n-1}y_{i+1},T^{n}y_{i+1}))\}\;\;\;\;\;\vdots \leq \mu^{n}\rho (y_{i},y_{i+1}).$$

Using inequality (3.4) in (3.3)

$$\rho (x,x^{⁎})\leq \mu^{n}\Sigma_{i=0}^{k-1}\rho (y_{i},y_{i+1})\to 0,\;\text{as}\;n\to \infty ,$$

a contradiction. So $\rho (x,x^{⁎})=0$, that is, $x=x^{⁎}$. □

Remark 3.1 The range space T(X) of X is supposed to be $R^{s}$-connected in Theorem 3.1 rather than the non-emptiness of the collection of paths within a binary relation R to establish the singularity of the fixed point.

Next, we proceed to establish the corollary derived from Theorem 3.1, wherein we substitute the $R^{s}$-connectedness of T(X) with different conditions.

Corollary 3.1 *If* (*v*) *of* Theorem 3.1 *is substituted by one of the subsequent suppositions*

$\left(x_{1}\right)$ $R{|}_{T(X)}$*is complete.*
$\left(x_{2}\right)$ $\Gamma (x,y,R^{s})\neq \phi$*.*
$\left(x_{3}\right)$ T(X)*is*$R^{s}$*-directed.*

*Then, the deduction of* Theorem 3.1 *holds.*

Proof

(1) If $R{|}_{T(X)}$ is complete, then $[x,y]\in R{|}_{T(X)}$, conveys that {x,y}constitutes a path of length 1 in the context of $R^{s}{|}_{T(X)}$ connecting *x* to *y* for x,y∈T(X). Thus T(X) is $R^{s}$-connected, and Theorem 3.1 deduces the result.
(2) If $\Gamma (x,y,R^{s})\neq \phi$, then for x,y∈T(X), a path $\left\{z_{0},z_{1},z_{2},\ldots ,z_{k}\right\}$ (say) of length *k* in the context of $R^{s}$ connecting *x* to *y* and $z_{0}=x,\;z_{k}=y$. Hence, T(X) is $R^{s}$-connected and Theorem 3.1 deduces the desired outcome.
(3) If T(X) is $R^{s}$-directed, then for x,y∈T(X), there exist y∈X and $(x,z),(y,z)\in R^{s}$, that is, {x,z,y} constitutes a path of length 2 in the context of $R^{s}$ connecting *x* to *y*. Thus, T(X) is $R^{s}$-connected and Theorem 3.1 deduces the desired outcome. □

The following examples substantiate the main conclusion.

Example 3.4 Consider the partial metric described on a set X=[0,10) as ρ(x,y)=max⁡{x,y}+|x−y|. Let a relation R on X be

R(x,y)={(0,0),(0,3),(3,0),(3,3),(1,8),(3,8),(8,8)},

whose symmetric closure is

$$R^{s}(x,y)=\left\{(0,0),(0,3),(3,0),(3,3),(0,8),(8,0),(3,8),(8,3),(8,8)\right\}.$$

Next, consider a self-map T on the set X=[0,10) as

$$Tx=\left\{ \begin{array}{cc} 0, & x\in [0,3]\\ 3, & x\in (3,8]\\ 8, & x\in (8,10) \end{array}\right..$$

T(X)={0,3,8}. (X,ρ) is not complete space however, (X,ρ,R) is R-complete. Here X[T,R]≠ϕ. Evidently, T is not continuous but R continuous. Also, R is *ρ*-self-closed. Our suppositions of Theorem 3.1 are substantiated for $\mu \in [\frac{3}{8},1)$ and a self-map T possesses a unique fixed point at x=0. Additionally, we have a sequence $\left\{x_{n}\right\}\subseteq X,\;x_{n}=\{0,3,0,3,3,0,0,0,\ldots ,0\},\;n\in N$, converging to 0.

Example 3.5 Consider the partial metric described on a set X=[0,∞) as ρ(x,y)=max⁡{x,y}+|x−y| and a relation R on X as

R(x,y)={(x,y):x≥y},

whose symmetric closure is

$$R^{s}(x,y)=\left\{(x,y):x\leq y\right\}.$$

Next consider a self-map T on a set X=[0,∞) as

$$Tx=\left\{ \begin{array}{cc} 0, & x\in [0,3]\\ x-3, & x\in (3,8]\\ 5, & x\in (8,\infty ) \end{array}\right..$$

T(X)=[0,5]. (X,ρ,R) is R-complete. Here X[T,R]≠ϕ and R is T-closed. Evidently, T is R continuous and R is *ρ*-self-closed. Our suppositions of Theorem 3.1 are substantiated for $\mu =\frac{5}{8}$ and T possesses a unique fixed point at x=0. Additionally, we have a sequence $\left\{x_{n}\right\}\subseteq X,\;x_{n}=\{\frac{n}{n^{2}+1}\},\;n\in N$, converging to 0.

Example 3.6 Let $\rho (x,y)=\max\{x,y\}+\frac{\left|x-y\right|}{1+|x-y|}$ be a partial metric on a set X=[0,10). Noticeably, (X,ρ) is neither complete nor a standard metric space. Let relation R on X be described as

R(x,y)={(0,0),(0,1),(1,0),(1,2),(4,0),(0,4),(4,4),(3,0),(4,5),(5,0),(0,5),(5,5),(6,0),(0,6),(6,6),(0,7),(7,6),(7,8),(8,0),(0,8),(8,8),(9,0),(9,8)},

whose symmetric closure is

$$R^{s}(x,y)=\{(0,0),\;(0,1),\;(1,0),\;(1,2),\;(4,0),\;(0,4),\;(4,4),\;(3,0),\;(0,3),\;(4,5),\;(5,4),\;(5,0),\;(0,5),\;(5,5),\;(6,0),(0,6),\;(6,6),\;(0,7),\;(7,0),\;(7,6),\;(6,7),\;(7,8),\;(8,7),\;(8,0),\;(0,8),\;(8,8),\;(9,0),\;(0,9),\;(9,8),\;(8,9),\;(2,1)\}.$$

Now, consider a self-map T on a set X=[0,10) as

$$T(x)=\left\{ \begin{array}{cc} 0, & x\in [0,1]\\ 1, & x\in (1,5]\\ 5, & x\in (5,7)\\ 6, & x\in [7,10) \end{array}\right..$$

T(X)={0,1,5,6}. (X,ρ,R) is R-complete. Here X[T,R]≠ϕ and R is T-closed. Evidently, T is not continuous. Consider an R-preserving sequence $\left\{x_{n}\right\}$ so that $x_{n}⟶x$ and $(x_{n},x_{n+1})\in R{|}_{T(X)},\;n\in N_{0}$. Here $\left(x_{n},x_{n+1})\in \{(0,0),\;(1,0),\;(0,1),(1,1),\;(4,0),\;(0,4),\;(4,4),\;(5,0),\;(0,5),\;(5,5),\;(6,0),\;(0,6),\;(6,6),\;(8,0),\;(0,8),\;(8,8)\right\}$ and $(x_{n},x_{n+1})\notin \{(1,2).\;(3,0),\;(4,5),\;(7,6),\;(7,8),\;(9,0),(9,8)\},\;n\in N_{0}$. Hence, $\left\{x_{n}\}\subset \{0,\;1,\;2,\;5,\;6,\;8\right\}$. $\lim_{n\to \infty }x_{n}=x\in \{0,\;1,\;2,\;5,\;6,\;8\}$ for each R-preserving sequence and a subsequence $\left\{x_{n_{k}}\right\}$ of sequence $\left\{x_{n}\right\}$ so that $x_{n_{k}}\to x$ and $[x_{n_{k}},x]\in R$, k∈N. Thus, R is *ρ*-self-closed and T(X) is $R^{s}$-connected. The suppositions of Theorem 3.1 are substantiated for $\mu =\frac{8}{13}$ and a self-map T possesses a unique fixed point at x=0. Additionally, we have a sequence $\{x_{n}\}\subseteq X$, $x_{n}=\left\{0,1,0,5,6,0,0,\ldots ,0\right\}$, which converges to 0.

Remark 3.2

(1) It is fascinating to observe that, Theorem 3.1 is an improvement and enhancement of Alam and Imdad [2], Alnasera et al. [4], Banach [8], Chatterjea [9], Ćirić [10]-[11], Hardy Rogers [12], Kannan [13], Nieto and Rodríguez-López [22], and Reich [24] to a partial metric space endowed with a binary relation. We have substituted the continuity of an underlying map by R-continuity, *ρ*-self-closedness of R by *ρ*-self-closedness of $R{|}_{Y}$, where Y⊆X and nonemptiness of the class of paths in R by $R^{s}$-connectedness of range space.
(2) Apparently, (X,ρ,R) is not complete in Example 3.4, Example 3.6 and T is discontinuous.
(3) Actually, binary relation R is reflexive, nonsymmetric, and near-order. Hence, R is neither strict order nor tolerance nor simple order.

Now, we establish a relation theoretic version of Hardy-Roger [12] in (X,ρ,R).

Theorem 3.2 *If*(iv)*of*Theorem 3.1*is substituted by*${\left(iv\right)}^{\prime }$*,*

${\left(iv\right)}^{\prime }$ *there exists*α+β+γ+δ+η<1*,*α,β,γ,δ,η≥0*, so that*
  (3.5) $$\rho (Tx,Ty)\leq \Delta_{1}(x,y),\;(x,y)\in R,$$
  *where,*$\Delta_{1}(x,y)=\alpha \rho (x,y)+\beta \rho (x,Tx)+\gamma \rho (y,Ty)+\delta \rho (x,Ty)+\eta \rho (y,Tx)$*.*

*Then, also the deduction of*Theorem 3.1*is true.*

Proof Examine a Picard sequence $\left\{x_{n}\right\}\subseteq X$ so that $x_{n+1}=Tx_{n}$, $n\in N_{0}$, and starting from an initial point $x_{0}\in X[T,R]$ as X[T,R]≠ϕ. Since $(x_{0},Tx_{0})\in R$ and R is T-closed, so, $(Tx_{0},T^{2}x_{0}),(T^{2}x_{0},T^{3}x_{0}),\ldots ,(T^{n}x_{0},T^{n+1}x_{0})\in R$. Hence, $(x_{n},x_{n+1})\in R$, $n\in N_{0}$, that is, the sequence $\left\{x_{n}\right\}$ is an R-preserving.

Using inequality (3.5),

$$\rho (x_{n},x_{n+1})=\rho (Tx_{n-1},Tx_{n})\leq \Delta_{1}(x_{n-1},x_{n}),\;n\in N_{0},$$

where,

$$\Delta_{1}(x_{n-1},x_{n})=\alpha \rho (x_{n-1},x_{n})+\beta \rho (x_{n-1},Tx_{n-1})+\gamma \rho (x_{n},Tx_{n})+\delta \rho (x_{n-1},Tx_{n})+\eta \rho (x_{n},Tx_{n-1})=\alpha \rho (x_{n-1},x_{n})+\beta \rho (x_{n-1},x_{n})+\gamma \rho (x_{n},x_{n+1})+\delta \rho (x_{n-1},x_{n+1})+\eta \rho (x_{n},x_{n})\leq (\alpha +\beta )\rho (x_{n-1},x_{n})+\gamma \rho (x_{n},x_{n+1})+\delta (\rho (x_{n-1},x_{n})+\rho (x_{n},x_{n+1})-\rho (x_{n},x_{n}))+\eta \rho (x_{n},x_{n})\leq (\alpha +\beta +2\delta +\eta )\rho (x_{n-1},x_{n})+(\gamma +\delta )\rho (x_{n},x_{n+1}).$$

Now, we have

(3.6) $$(1-\gamma -\delta )\rho (x_{n},x_{n+1})\leq (\alpha +\beta +2\delta +\eta )\rho (x_{n-1},x_{n})\rho (x_{n},x_{n+1})\leq \frac{\alpha +\beta +2\delta +\eta }{1-\gamma -\delta }\rho (x_{n-1},x_{n})=\lambda \rho (x_{n-1},x_{n}),$$

assuming, $\lambda =\frac{\alpha +\beta +2\delta +\eta }{1-\gamma -\delta }<1$. Now, by induction on *n* and using (3.6)

(3.7) $$\rho (x_{n},x_{n+1})\leq \lambda^{n}\rho (x_{0},x_{1})\to 0,\;\text{as}\;n\to \infty .$$

Next, following the same steps as used in Theorem 3.1 yields that x∈X so that $x_{n}⟶x$.

If T is R-continuous, then $x_{n+1}=Tx_{n}\to Tx$. So Tx=x, hence *x* is a fixed point of T.

Consider R to be *ρ*-self-closed, then for an R-preserving sequence $\left\{x_{n}\right\}$ in X as $x_{n}⟶x$, we have a subsequence $\left\{x_{n_{k}}\right\}$ of $\left\{x_{n}\right\}$ satisfying $[x_{n_{k}},x]\in R$, $k\in N_{0}$ and $x_{n_{k}}⟶x$. Now, suppose x≠Tx.

$$\rho (x,Tx)\leq \rho (x,Tx_{n_{k}})+\rho (Tx_{n_{k}},Tx)-\rho (Tx_{n_{k}},Tx_{n_{k}})=\rho (x,x_{n_{k+1}})+\alpha \rho (x_{n_{k}},x)+\beta \rho (x_{n_{k}},Tx_{n_{k}})+\gamma \rho (x,Tx)+\delta \rho (x_{n_{k}},Tx)+\eta \rho (x,Tx_{n_{k}})-\rho (x_{n_{k+1}},x_{n_{k+1}})=\rho (x,x_{n_{k+1}})+\alpha \rho (x_{n_{k}},x)+\beta \rho (x_{n_{k}},x_{n_{k}+1})+\gamma \rho (x,Tx)+\delta \rho (x_{n_{k}},Tx)+\eta \rho (x,x_{n_{k}+1})-\rho (x_{n_{k+1}},x_{n_{k+1}})\to \rho (x,x)+\alpha \rho (x,x)+\beta 0++\gamma \rho (x,Tx)+\delta \rho (x,Tx)+\eta \rho (x,x)-\rho (x,x),\;\;\text{as}\;\;n\to \infty ,(1-\gamma -\delta )\rho (x,Tx)<(\alpha +\eta )\rho (x,x)\rho (x,Tx)<\frac{\alpha +\eta }{1-\gamma -\delta }\rho (x,x)<\rho (x,x),$$

a contradiction. Hence, Tx=x and *x* is a fixed point of a self-map T.

Let F(T) be a singleton set then the result is obvious. Suppose, *x* and $x^{⁎}$ are both fixed points of T, that is, Tx=x and $Tx^{⁎}=x^{⁎}$. So

$$x,\;x^{⁎}\in T(X)\;\;\text{and}\;\;T^{n}x=x,\;\;T^{n}x^{⁎}=x^{⁎},\;\;n\in N_{0}.$$

As, T(X) is $R^{s}$-connected, we have a path $\left\{y_{0},\;y_{1},\;y_{2},\;\ldots ,\;y_{k}\right\}$ of length *k* within the context of $R^{s}$ starting from *x* and ending at $x^{⁎}$ satisfying $y_{0}=x,\;y_{k}=x^{⁎}$ so that $(y_{i},y_{i+1})\in R^{s}$, 0≤i≤k−1. Because R is T-closed, $(T^{n}y_{i},T^{n}y_{i+1})\in R^{s}$, $n\in N_{0}$. So

(3.8) $$\rho (x,x^{⁎})=\rho (T^{n}y_{0},T^{n}y_{k})\leq \Sigma_{i=0}^{k}\rho (T^{n}y_{i},T^{n}y_{i+1})-\Sigma_{i=1}^{k-1}\rho (T^{n}y_{i},T^{n}y_{i})\leq \Sigma_{i=0}^{k}\rho (T^{n}y_{i},T^{n}y_{i+1}).$$

$$\rho (T^{n}y_{i},T^{n}y_{i+1})\leq \alpha \rho (T^{n-1}y_{i},T^{n-1}y_{i+1})+\beta \rho (T^{n-1}y_{i},T^{n}y_{i+1})+\gamma \rho (T^{n}y_{i+1},T^{n}y_{i})\;\;\;\;\;\delta \rho (T^{n-1}y_{i},T^{n}y_{i})+\eta \rho (T^{n-1}y_{i+1},T^{n}y_{i+1})$$

(3.9) $$\rho (y_{n+i},y_{n+i+1})\leq \alpha \rho (y_{n+i-1},y_{n+i})+\beta \rho (y_{n+i-1},y_{i+n+1})+\gamma \rho (y_{n+i+1},y_{n+i})\;\;\;\;\;\delta \rho (y_{n+i-1},y_{n+i})+\eta \rho (y_{n+i},y_{n+i+1})(1-\beta -\gamma -\eta )\rho (y_{n+i},y_{n+i+1})\leq (\alpha +\beta +\delta )\rho (y_{n+i-1},y_{n+i})\rho (y_{n+i},y_{n+i+1})\leq \frac{\alpha +\beta +\delta }{1-\beta -\gamma -\eta }\rho (y_{n+i-1},y_{n+i})\rho (y_{n+i},y_{n+i+1})\leq \xi \rho (y_{n+i},y_{n+i+1})\;\;\;\;\;\vdots \leq \ldots =\xi^{n}\rho (y_{i},y_{i+1}),\;\text{where},\;\xi =\frac{\alpha +\beta +\delta }{1-\beta -\gamma -\eta }.$$

Using inequality (3.9) in (3.8)

$$\rho (x,x^{⁎})\leq \xi^{n}\Sigma_{i=0}^{k-1}\rho (y_{i},y_{i+1})\to 0,\;\text{as}\;n\to \infty ,$$

a contradiction. So $\rho (x,x^{⁎})=0$, that is, $x=x^{⁎}$. □

Continuing the same pattern, we now derive a corollary based on Theorem 3.2, wherein we substitute the $R^{s}$-connectedness of T(X) with alternative conditions.

Corollary 3.2 *If*$R^{s}$*-connectedness of*T(X)*in*Theorem 3.2*is substituted by one of the subsequent suppositions*

$\left(x_{1}\right)$ $R{|}_{T(X)}$*is complete.*
$\left(x_{2}\right)$ T(X)*is*$R^{s}$*-directed.*
$\left(x_{3}\right)$ $\Gamma (x,y,R^{s})\neq \phi$*.*

*Then, the deduction of*Theorem 3.2*remains valid.*

Proof The proof adopts a structure akin to that of Corollary 3.1. □

Corollary 3.3 *If*(iv)*of*Theorem 3.1*is substituted by*${\left(iv\right)}^{\prime \prime }$

${\left(iv\right)}^{\prime \prime }$ *there exists*μ∈[0,1)*, satisfying*
  (3.10) $$\rho (Tx,Ty)\leq \mu \Delta_{2}(x,y),$$
  *where*$\Delta_{2}(x,y)=\max\{\rho (x,y),\rho (x,Tx),\rho (y,Ty),\frac{1}{2}(\rho (x,Tx)+\rho (y,Ty),\frac{1}{2}(\rho (x,Ty)+\rho (y,Tx)\},(x,y)\in R$*.*

*Then, the deduction of*Theorem 3.1*remains valid.*

Proof The proof adopts a structure akin to that of Theorem 3.1. □

Remark 3.3

(*i*) If (*v*) of Corollary 3.3 is substituted by any one of the $\left(x_{1}\right)$, $\left(x_{2}\right)$, or $\left(x_{3}\right)$, then the above consequences hold.
(ii) Suitably choosing the values of constants α,β,γ,δ,η in Theorem 3.2, we achieve the extensions, improvements and generalizations of Banach [8], Chatterjea [9], Kannan [13], Reich [24], and others to a relational partial metric space.
(iii) In the above results if R=X×X, we get development and advancement of glorious conclusions in partial metric and ordered partial metric spaces, which are not even complete and the underlying self map is discontinuous. As a result, our conclusions extend, improve, and generalize the majority of glorious conclusions present in the literature.

We now provide two illustrative examples featuring discontinuous maps in a noncomplete partial metric space endowed with binary relation.

Example 3.7 Consider a partial metric on a set X=(5,15] as ρ(x,y)=max⁡{x,y}. Describe a binary relation R on X=(5,15] as

R={(x,y):x≥y},

whose symmetric closure is

$$R^{s}=\left\{(x,y):\;either\;x\leq y\;or\;y\geq x\right\}.$$

Consider a self-map T on a set X=(5,15] as $T(x)=\left\{ \begin{array}{cc} \frac{11}{2}, & x\in (5,8]\\ 6, & x\in (8,11]\\ \frac{13}{2}, & x\in (11,12]\\ 7, & x\in (12,15] \end{array}\right.$.

Here $T(X)=\{\frac{11}{2},\;6,\;\frac{13}{2},\;7\}$. Clearly, (X,ρ) is not complete but (X,ρ,R) is R-complete. Here X[T,R]≠ϕ and R is T-closed. Evidently, T is not continuous but R is *ρ*-self-closed. Our suppositions of Theorem 3.2 are substantiated for $\alpha =\beta =\gamma =\frac{6}{20},\;\delta =\eta =\frac{1}{32}$ and T possesses a unique fixed point at $x=\frac{11}{2}$. Additionally, we have a sequence $\left\{x_{n}\right\}\subseteq X,\;x_{n}=\frac{11n}{2n+1},\;n\in N$, converging to $\frac{11}{2}$.

Example 3.8 Consider a partial metric on a set X=(0,20] as ρ(x,y)=max⁡{x,y}+|x−y|. If a binary relation represented as R on X as

R(x,y)={(5,10),(10,5),(10,10),(10,15),(15,10),(15,15),(9,12),(13,17),(20,20)},

whose symmetric closure is

$$R^{s}(x,y)=\{(5,10),\;(10,5),\;(10,10),\;(10,15),\;(15,10),\;(15,15),\;(9,12),\;(12,9),\;(13,17),\;(17,13),\;(20,20)\}.$$

Now, let a self-map T on a set X=(0,20] be

$$T(x)=\left\{ \begin{array}{cc} 5, & x\in (0,15)\\ 10, & x\in (15,19)\\ 20, & x\in [19,20] \end{array}\right..$$

Here T(X)={5,10,20}⊂Q=P. Clearly, (X,ρ) is not complete but (X,ρ,R) is complete. Here X[S,R]≠ϕ. It is evident that T is not continuous but R is *ρ*-self-closed with respect to the partial metric *ρ*. Hence, our suppositions of Theorem 3.2 are substantiated for $\alpha =\beta =\gamma =\frac{19}{20},\;\delta =\eta =\frac{15}{16}$ except (vi) and T has two fixed points at 5 and 20. Further, there exists sequences $x_{n}=\{10,5,10,5,10,5,5,5,\ldots ,5\}$ and $y_{n}=\{10,20,10,10,20,20,\ldots ,20\},n\in N$, converging to 5 and 20 respectively.

Remark 3.4

(*i*) A self-map T is assumed to be R-continuous rather than continuous and R is assumed to be *ρ*-self-closed in established Theorems and corollaries (see also Example 3.4, Example 3.6 - 3.8) and we established that continuity does not have a substantial impact on the presence of a fixed point in our conclusions.
(ii) Example 3.8 establishes that any one of the $R^{s}$-directedness or nonemptiness of collection of paths in R or $R^{s}$-connectedness of range space is necessary for the survival of uniqueness of fixed point. Alternatively, a self-map may have more than one fixed point.

Remark 3.5 It is fascinating to observe that relation-theoretic nonlinear contractions are relatively weaker as compared to celebrated standard contractions because these are valid only for the points in the underlying binary relation (see, Example 3.4, Example 3.8). For establishing the relation theoretic fixed point for set-valued maps in partial metric space, one may allude to Tomar et al. [26] and for single-valued maps within F-metric space, *b*-metric-like space, and via simulation function approach in metric space, refer to Tomar and Joshi [27], Tomar et al. [28]-[29], Antal et al. [5], and references therein.

Remark 3.6 Although partial metric spaces may seem counterintuitive compared to traditional metric spaces, they offer significant insights in functional analysis and fixed-point theory. By allowing non-zero self-distances, partial metric spaces facilitate a richer analysis of convergence and continuity, proving particularly useful in applications like elliptic boundary value problems and integral equations. Our work demonstrates their practical utility and potential to complement existing theories.

Remark 3.7 Relational partial metric spaces extend traditional metrics by allowing flexible distance definitions that prioritize relationships over strict numerical distances. This framework is particularly advantageous in fields like computer science and network theory, where standard metrics may be restrictive or fail to adequately represent the underlying relationships among elements. While some classical results may not directly apply to relational partial metric spaces, this encourages a re-evaluation and adaptation of foundational concepts, paving the way for new results that address complex relationships across various domains. This is especially beneficial when the relationships among points are more significant than the distances themselves, making relational partial metric spaces valuable in contexts where standard assumptions of distance do not hold, such as in certain optimization problems or when dealing with abstract mathematical constructs.

## 4 Applications

Now, we explore the practical implications of our research, focusing on a solution to an elliptic boundary value problem and an integral equation in partial metric space associated with binary relation. The resolution of elliptic boundary value problems and integral equations in such spaces is a testament to the power and adaptability of mathematical theory in addressing challenges at the forefront of contemporary research.

### 4.1 Resolving elliptic boundary value problem

An elliptic boundary value problem is a type of differential equation common in physics and engineering, particularly in steady-state heat conduction, fluid flow, and elasticity. It involves a second-order partial differential equation defined over a specific domain with boundary conditions such as Dirichlet, Neumann, or Robin. The term “elliptic” indicates that the differential operator satisfies conditions ensuring well-posed solutions, typically involving positive eigenvalues.

Elliptic boundary value problems are crucial in mathematical physics, modeling stable phenomena that do not depend on time. Solving an elliptic boundary value problem provides a function that satisfies the governing equation and the specified boundary conditions, offering insights into the physical behavior of the system. A classic example is the steady-state heat distribution in a metal plate, modeled by the Laplace equation $\nabla^{2}T=0$, with boundary conditions defining the temperature along the edges. Overall, elliptic boundary value problems are fundamental in applied mathematics to address various physical and engineering challenges.

Consider C[I,R] to symbolize the collection of continuous functions on I=[0,1] and X=C[I,R]. We first solve an elliptic boundary value problem as an application to our Theorem 3.1.

Theorem 4.1 *In the context of the given elliptic boundary value problem*

(4.1) $$-\frac{d^{2}x}{dt^{2}}=\psi (t,x(t)),\text{with}\;x(0)=0,x(1)=0,$$

*assume*ψ:[0,1]×R⟶R*to be a continuous and increasing function and*t∈I*. In this case, the associated Green function linked with the given elliptic boundary value problem*(4.1)*is:*

$$G(t,x)=\left\{ \begin{array}{c} (1-t)x,\;\;0\leq x\leq t\leq 1\\ (1-x)t,\;\;0\leq t\leq x\leq 1. \end{array}\right.$$

*Define*$\left\|x\|=\max_{t\in [0,1]}|x(t)\right|$*and a partial metric*ρ:C(I)→C(I)*as*$\rho (x,y)=\left\{ \begin{array}{cc} \|x-y\|, & \|x\|,\;\|y\|\leq 1\\ \|x-y\|+\zeta , & \;\text{otherwise} \end{array}\right.,\;\zeta \geq 1$*. So*(X,ρ)*is an*R*-complete partial metric space. Suppose map*T:X→X*is defined as*

(4.2) $$Tx(t)=\int_{0}^{1}G(t,x)\psi (x,x(x))dx,\;for\;t\in I.$$

*If there is a value*κ≥0*such that*

(4.3) $$0\leq \|\psi (t,x(t))-\psi (t,y(t))\|\leq \kappa \frac{\|x(t)-y(t)\|+\zeta }{1+\|x(t)-y(t)\|},$$

x(t),y(t)∈X*with*x(t)≥y(t)*then problem*(4.1)*possesses a unique solution*$x^{⁎}$*in*X*.*

Proof Apparently, x∈X is the solution of (4.1) if and only if it is the solution of (4.2). Consider a binary relation represented as

R={(x,y)∈X×X:x(t)y(t)≥0with(x−y)(t)≥0,∀t∈I}.

(i) Assuming an R-preserving Cauchy sequence $\left\{x_{n}(t)\right\}$ such that $\left\{x_{n}(t)\right\}$ converges to {x(t)}, we observe that $x_{n}(t)x_{n+1}(t)\geq 0$ and $x_{n}(t)\geq x_{n+1}(t)$ for *t* in the interval *I* and *n* in the set of natural numbers $N_{0}$. We have two scenarios: either $x_{n}(t)$ is positive or $x_{n}(t)$ is negative. If $x_{n}(t)\geq 0$, it implies that for *t* in the interval *I*, there is a sequence of positive real numbers approaching the limit $x_{(}t)$. Consequently, x(t)≥0, implying that $\left(x_{n}(t),x(t)\right)$ belongs to R for *t* in the interval *I* and $n\in N_{0}$. That is, R is *ρ*-self-closed.
(ii) Assume (x,y)∈R, that is, x(t)≥y(t). Because *ψ* is increasing, so ψ(t,x(t))≥ψ(t,y(t)) and G(t,x)≥0, (t,x)∈I×I, and
  $$Tx(t)=\int_{0}^{1}G(t,x)\psi (x,x(x))dx\geq \int_{0}^{1}G(t,x)\psi (x,y(x))dx=Ty(t),$$
  ⟹ (Tx(t),Ty(t))∈R, that is, R is T-closed.
  Further, for x(t)≥0, Tx(t)≥0, that is, (x(t),Tx(t))∈R.
  Hence, X[T,R]≠ϕ.
(iii) For ‖x‖,‖y‖≤1, (x,y)∈R,
  $$\rho (Tx(t),Ty(t))=\|Tx(t)-Ty(t)\|=\|\int_{0}^{1}G(t,x)\psi (x,x(x))dx-\int_{0}^{1}G(t,x)[\psi (x,y(x))dx\|\leq \|\int_{0}^{1}G(t,x)\frac{x(x)-y(x)\|}{1+\|x(x)-y(x)\|}dx\leq \|x(t)-y(t)\|\int_{0}^{1}G(t,x)dx=\left|x(t)-y(t)\right|\int_{0}^{t}(1-t)xdx+\int_{t}^{1}(1-x)tdx\leq \left|x(t)-y(t)\right|(\frac{1}{8})=\mu \|x(t)-y(t)|\leq \mu \max\{\rho (x,y),\rho (x,Tx),\rho (y,Ty),\frac{1}{2}(\rho (x,Ty)+\rho (y,Tx))\},\;\mu =\frac{1}{8}\in [0,1).$$

T(X) is $R^{s}$-connected. So, the suppositions of Theorem 3.1 are substantiated and T possesses a unique fixed point, that is, an elliptic boundary value problem (4.1) possesses exactly one solution. □

### 4.2 Resolving integral equation

The formulation and analysis of integral equations in relational partial metric spaces require a delicate balance between abstract functional analysis and concrete computational methods. Integral equations, another facet of this intricate landscape, add a layer of complexity to the problem-solving process. Now, we apply our Theorem 3.2 to solve an integral equation.

Theorem 4.2 *Suppose the integral equation*

(4.4) $$x(t)=\int_{0}^{1}K(t,s,x(s))ds+f(t)=A_{x}(t)+f(t),$$

*where,*K:[0,1]×[0,1]×R→R*is continuous. Consider*$\left\|x\|=\max_{t\in [0,1]}|x(t)\right|$*and a partial metric*ρ:C(I)→C(I)*as*

$$\rho (x,y)=\left\{ \begin{array}{cc} \|x-y\|\sqrt{2}e^{\tan^{-1}\varpi }, & \|x\|,\;\|y\|\leq 1\\ \|x-y\|\sqrt{2}e^{\tan^{-1}\varpi }+\zeta , & \;\text{otherwise} \end{array}\right.,\;\zeta \geq 1.$$

*So*(X,ρ)*is an*R*-complete partial metric space. Suppose map*T:X→X*is defined as*

(4.5) $$Tx(t)=\int_{0}^{1}K(t,s,x(s))ds+f(t),\;for\;t\in I.$$

(4.6) $$\|Tx(t)-Ty(t)\|\sqrt{2}e^{\tan^{-1}\varpi }\leq J(x(t),y(t))+L(x(t),y(t)),$$

*where,*

$$J(x(t),y(t))=\|x(t)-y(t)\|\sqrt{2}e^{\tan^{-1}\varpi }$$

*and*

$$L(x(t),y(t))=\frac{\|A_{x}(t)+f(t)-x(t)\|\;\|A_{y}(t)+f(t)-y(t)\|\sqrt{2}e^{\tan^{-1}\varpi }}{1+\|A_{x}(t)+f(t)-x(t)\|\sqrt{2}e^{\tan^{-1}\varpi }},$$

x(t),y(t)∈X*with*x(t)≥y(t)*then problem*(4.4)*possesses a unique solution*$x^{⁎}$*in*X*.*

Proof Apparently, x∈X is the solution of (4.4) if and only if it is the solution of (4.5). Describe a binary relation

R={(x,y)∈X×X:x(t)y(t)≥0with(x−y)(t)≥0,∀t∈I}.

(i) For *t* in the interval *I* and *n* in the set of natural numbers $N_{0}$, assume an R-preserving Cauchy sequence $\left\{x_{n}(t)\right\}$ and $x_{n}(t)⟶x(t)$. We observe that $x_{n}(t)x_{n+1}(t)\geq 0$ and $x_{n}(t)\geq x_{n+1}(t)$. We have two scenarios: either $x_{n}(t)$ is positive or $x_{n}(t)$ is negative. If $x_{n}(t)\geq 0$, therefore, for t∈I, we observe a sequence of positive real numbers converging to x(t). Consequently, x(t)≥0, that is, $(x_{n}(t),x(t))\in R$, $n\in N_{0}$. So, R is *ρ*-self-closed.
(ii) If (x,y)∈R, that is, x(t)≥y(t).
  $$Tx(t)=\int_{0}^{1}K(t,s,x(s))ds+f(t)\geq \int_{0}^{1}K(t,s,y(s))ds+f(t)=Ty(t),$$
  ⟹ (Tx(t),Ty(t))∈R, that is, R is T-closed.
  Further, for x(t)≥0, Tx(t)≥0, that is, (x(t),Tx(t))∈R.
  Hence, X[T,R]≠ϕ.
(iii) If (x,y)∈R and ‖x‖,‖y‖≤1, then
  $$\rho (Tx(t),Ty(t))=\|Tx(t)-Ty(t)\|\sqrt{2}e^{\tan^{-1}\varpi }=\|J(x(t),y(t))+L(x(t),y(t))\|\leq \|x(t)-y(t)\|\sqrt{2}e^{\tan^{-1}\varpi }+L(x(t),y(t))\leq \|x(t)-y(t)\|\sqrt{2}e^{\tan^{-1}\varpi }+\|A_{y}(t)+f(t)-y(t)\|=\rho (x(t),y(t))+\rho (y(t),Ty(t))=\Delta_{1}(x,y).$$

T(X) is $R^{s}$-connected and the suppositions of Theorem 3.2 are substantiated and T has a unique fixed point, that is, an integral equation (4.4) has exactly one solution. □

Remark 4.1 To illustrate the applicability of relational partial metric spaces in solving elliptic boundary value problems and integral equations, consider the integral equation defined in Equation (4.4) and implement an iterative scheme based on our theoretical results, utilizing the relational partial metric structure to ensure convergence, which illustrates that the iterative method converges to a unique solution for various choices of **K** and f. The behavior of the solution aligns well with our theoretical expectations, confirming the efficacy of relational partial metric spaces in analyzing integral equations.

## 5 Conclusion

In conclusion, this paper demonstrates the significant potential of relational partial metric spaces in advancing functional analysis and fixed point theory. We have established the existence of a unique fixed point for Ćirić contraction [10]-[11] and Hardy-Roger contraction [12] within a relational partial metric space, particularly in the context of non-zero self-distance. We offer a versatile framework that broadens and refines traditional approaches, although it may require careful consideration of the underlying relational metrics. The proposed method replaces conventional continuity and completeness requirements with R-analogs, resulting in a more flexible approach with applications to solving elliptic boundary value problems and integral equations. This approach is particularly advantageous in scenarios where classical approaches may fall short, such as in settings involving relational structures or non-zero self-distances. Overall, our findings not only address the limitations of classical approaches but also pave the way for future research and cross-disciplinary applications, enriching existing theories and inspiring novel methodologies in various fields.

## CRediT authorship contribution statement

**Meena Joshi:** Writing – original draft, Methodology, Investigation, Conceptualization. **Anita Tomar:** Writing – review & editing, Investigation, Conceptualization. **Mohammad Sajid:** Writing – review & editing, Funding acquisition, Conceptualization. **S.K. Padaliya:** Methodology, Conceptualization.

## Declaration of Competing Interest

The authors declare the following financial interests/personal relationships which may be considered as potential competing interests: Mohammad Sajid reports financial support was provided by 10.13039/501100007414Qassim University. If there are other authors, they declare that they have no known competing financial interests or personal relationships that could have appeared to influence the work reported in this paper.

## Data Availability

No underlying data were collected or produced in this study.
